# Supplementary material for: Bitter Taste Receptor T2R14 and Autophagy Flux in Gingival Epithelial Cells
Source: Cells. 2024 Mar 17;13(6):531. doi: 10.3390/cells13060531 (PMC10969610; doi:10.3390/cells13060531)
Supplement: Supplementary file 1 [file cells-13-00531-s001.zip › cells-2853868-supplementary.pdf]

# Bitter taste receptor T2R14 and autophagy flux in gingival epithelial cells

**Nisha Singh[1,2], Ben Ulmer[1,2†], Manoj Medapati[1,2†], Christine Zhang[5], Robert Schroth[1,2,4], Saeid Ghavami[3*] and Prashen Chelikani[1,2,4*]**

[1]Manitoba Chemosensory Biology (MCSB) research group
[2]Dr. Gerald Niznick College of Dentistry, Rady Faculty of Health Sciences, University of Manitoba
[3] Research Institute of Oncology and Hematology, Cancer Care Manitoba-University of Manitoba, Winnipeg, Manitoba, Canada; Department of Human Anatomy and Cell Science, University of Manitoba College of Medicine, Winnipeg, Manitoba, Canada.
[4]Max Rady College of Medicine, Rady Faculty of Health Sciences, University of Manitoba
[5]University of Manitoba Flow Cytometry Core Facility, Rady Faculty of Health Sciences, University of Manitoba
†Equal contributions.
* These authors have senior co-authorship.
* Correspondence: email address: Prashen.Chelikani@umanitoba.ca, saeid.ghavami@umanitoba.ca, D319, Department of Oral Biology, 780 Bannatyne Avenue, University of Manitoba, Winnipeg, MB R3E 0W4. Canada, 204-789-3539 (phone), 204-789-3913 (fax)

Supplementary figures

S1

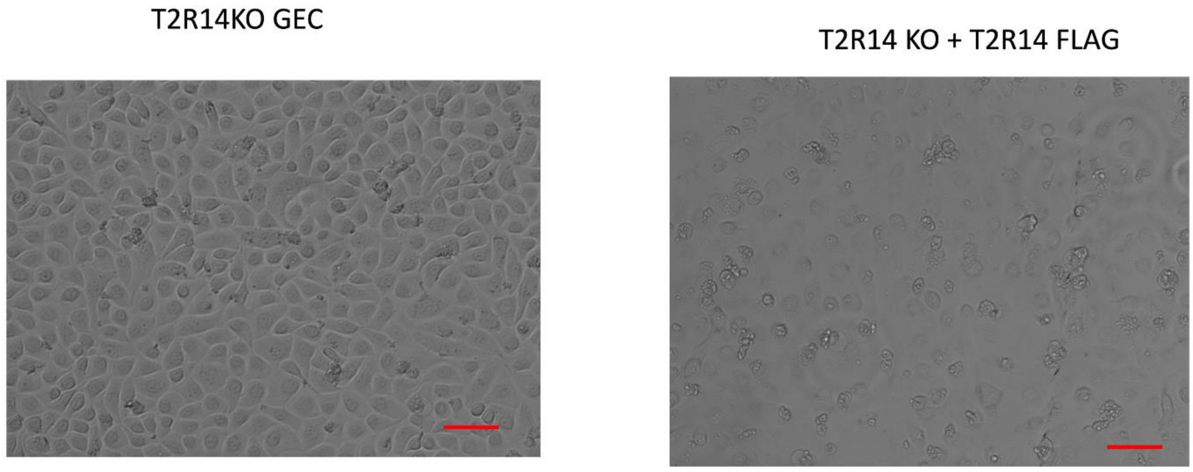


Figure S1: Light microscopy image showing GEC cell morphology after reintroduction of T2R14 FLAG in T2R14 KO GEC. Images were taken at 20x magnification.

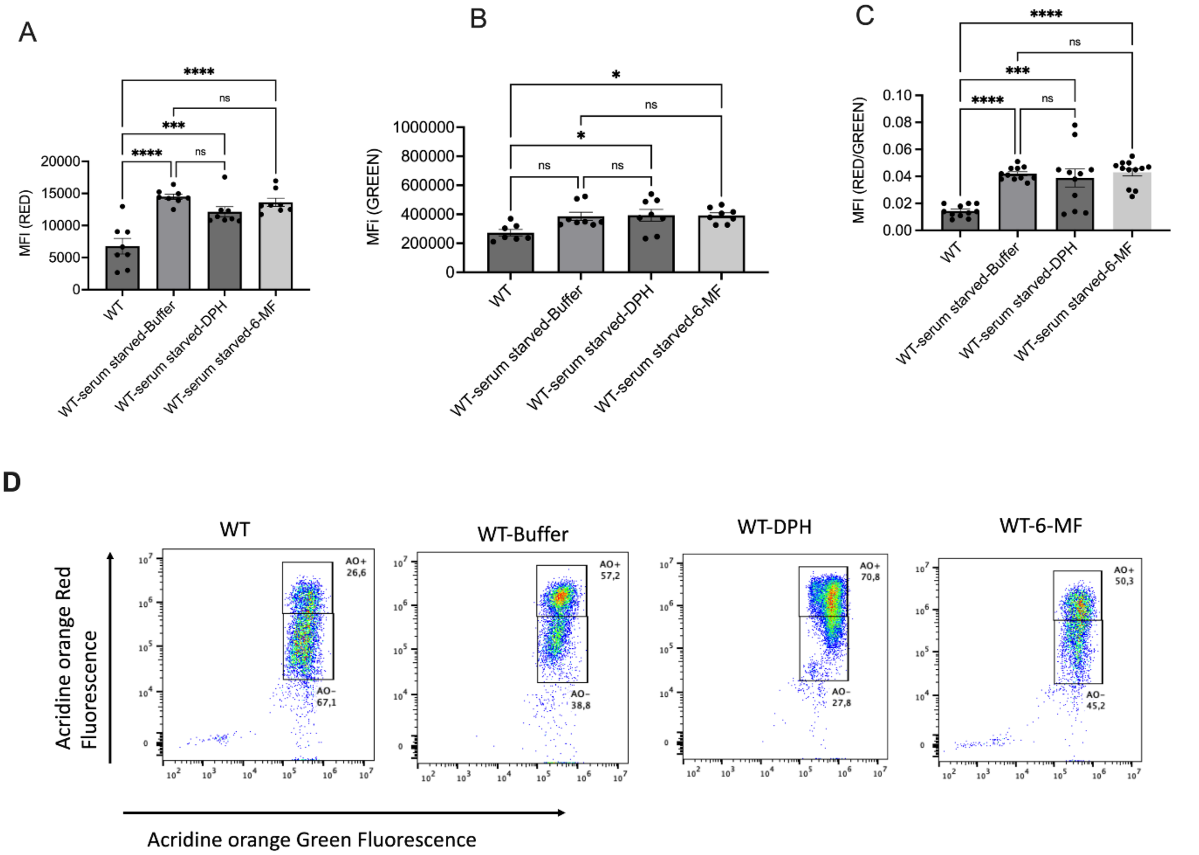


Figure S2: Acridine Orange staining of AVO for detecting autophagy flux in GEC upon treatments with DPH and 6-MF. **A-C**. Flow cytometry evaluation of red, green and red/green fluorescence ratio for detecting the autophagy flux in WT GEC under serum starved condition and with the selective agonist and antagonist treatments. The bar graphs were generated using graph pad prism 9.0. Statistical significance was calculated using One-way ANOVA with Bonferroni's post-hoc test *p<0.05, **p<0.01. The data represents SEM of 3 independent experiments. **D**. Acridine Orange staining of AVO for detecting autophagy flux in GEC. Representative raw traces showing flow cytometric detection of red and green fluorescence in acridine orange-stained WT GECs that were serum starved or treated with DPH (500 µM) and 6-MF (30 µM) for 24 hours for detecting the autophagy flux.
